# Successful treatment of refractory edema with traditional herbal medicine: A case report

**DOI:** 10.1097/MD.0000000000017551

**Published:** 2019-10-11

**Authors:** Gayoung Lee, Jung-Hyo Cho, Chang-Gue Son, Namhun Lee

**Affiliations:** a Department of Internal Medicine, Cheonan Korean Medicine Hospital of Daejeon University; b Liver and Immunology Research Center, Dunsan Korean Medicine Hospital of Daejeon University; c Department of Internal Medicine, Graduated School of Korean Medicine, University of Daejeon, Daejeon-si, Republic of Korea.

**Keywords:** cirrhosis, herbal medicine, refractory edema

## Abstract

**Rationale:**

Refractory edema is characterized by persistent swelling which does not react to diuretic use and sodium restriction. Traditional herbal medicine, Gwack Rhyung Tang and Chunggan extract effectively treated refractory lower limb edema caused by cirrhosis and improved liver function.

**Patient concerns:**

A 64-year-old male patient with a history of hypertension, diabetes mellitus, hepatic encephalopathy, and cellulitis presented lower limb edema which did not react to diuretics for more than 7 months.

**Diagnoses:**

Refractory edema caused by cirrhosis.

**Interventions:**

The patient was treated for 25 days using Gwack Rhyung Tang and Chunggan extract.

**Outcomes:**

Loss of body weight, decrease in circumferences of both lower limb and improvement of liver function biochemistry results are checked. There was no recurrence or aggravation of the condition up to 3 weeks of follow-up periods.

**Lessons:**

Traditional herbal medicine can be an effective alternative for refractory edema due to cirrhosis with improving liver function.

## 1 Introduction

Edema occurs in various disorders, such as heart failure, nephrotic syndrome, renal failure, cirrhosis, and cancer. The conventional treatment includes restricting dietary sodium and using diuretics, usually a loop diuretic, accompanied by specific treatments for each clinical disorder.^[1]^ Refractory edema is characterized by persistent swelling that does not respond to diuretic use and sodium restriction. Common causes include nonadherence to sodium or fluid restriction or the drug, reduced diuretic secretion, and deficiency of the kidney response to the drug.^[2]^ There are several approaches to refractory edema, including the use of different types of diuretic, lifestyle modification, and optimization of renal perfusion.^[3–5]^

Cirrhosis can cause edema, ascites, and pleural effusions, resulting from a loss of equilibrium between the plasma and extracellular fluid. Hepatorenal syndrome may worsen the fluid retention caused by low cardiac output and impaired renal perfusion.^[6,7]^ Diuretic resistance in patients with liver cirrhosis can be explained by the limited absorption of oral diuretics because of reduced intestinal motility and perfusion. Moreover, reduced splanchnic vasodilation causes the vasoconstriction of renal blood vessels, which results in limited diuretic secretion.^[2]^ Although sodium and water restriction are commonly recommended, this may cause loss of appetite and malnutrition. Considering the vulnerability of cirrhosis patients to the drugs used for treatment, toxicity due to high doses and complex prescriptions of diuretics can be fatal.^[8]^

In such cases, traditional herbal medicine (THM) is an alternative therapy. THM has been widely prescribed alone or in combination with conventional Western drugs for edema in South Korea. Here, we report a case of refractory lower limb edema with cirrhosis successfully treated with THM. This study followed the Case Report Guide line (CARE guidelines)^[9]^ with patient's informed consent and was approved by the Institutional Review Board of Daejeon University Korean medical hospital (DJUMC-2019-BM-1).

## 2 Patient information

A 64-year-old man with liver cirrhosis was admitted to the Korean medicine hospital on December 4, 2018, with a chief complaint of lower limb edema and pruritus involving his whole body for more than 7 months. Despite treatment with diuretics, his condition did not respond to the medication and his quality of life deteriorated. Therefore, he revisited our hospital and requested an alternative treatment. He had a history of hypertension and diabetes mellitus and 3 months before this admission, he was admitted to a local hospital to treat hepatic encephalopathy and cellulitis of the right leg.

## 3 Clinical findings and diagnostic assessment

**Figure 1:**
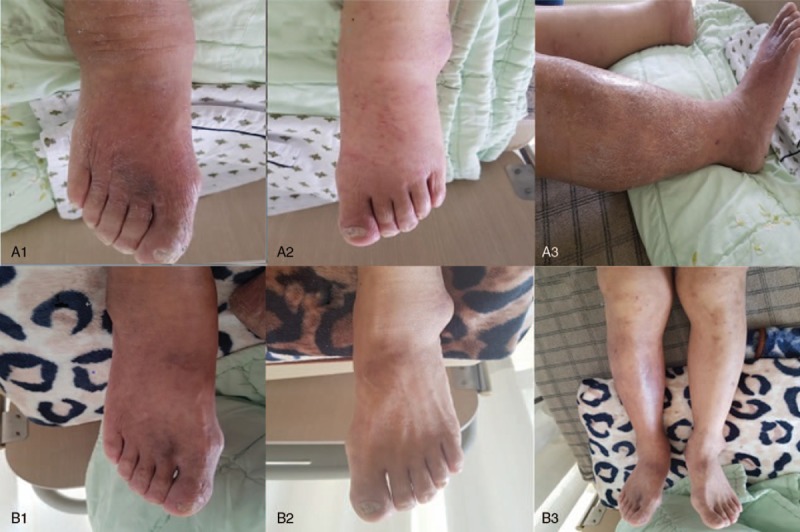
Clinical images showing the changes of both lower limb edema during the treatment period. (A) Both lower limbs, especially right ankle and calf showed obvious edema with heating sense and redness at admission day. (A-1: Right foot, A-2: Left foot, A-3: Right leg) (B) After 3 weeks after the start of the treatment, both ankle and feet slimmed down. (B-1: Right foot, B-2: Left foot, B-3: Both legs).

**Figure 2:**
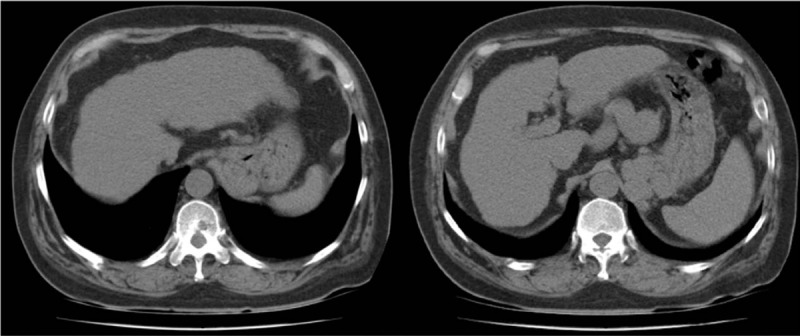
Abdomen CT on Dec 5, 2018. It revealed liver cirrhosis with atrophy of liver, fat deposition in liver, esophageal varix along lesser curvature of stomach and no ascites.

Both lower limbs were swollen, particularly the right leg, which caused difficulty walking (Fig. 1A). Computed tomography revealed liver cirrhosis with atrophy of the liver, fat deposition in the liver, an esophageal varix along the lesser curvature of the stomach, and splenomegaly (Fig. 2). Biochemical analyses indicated increases in aspartate aminotransferase (AST), alkaline phosphatase (ALP), gamma-glutamyl transferase (GGT), blood urea nitrogen (BUN), and creatinine (Cr) and decreases in total protein and albumin levels. Total bilirubin levels were elevated at 3.23 mg/dL (Table 1). We diagnosed refractory edema caused by cirrhosis with renal insufficiency.

**Table 1:**
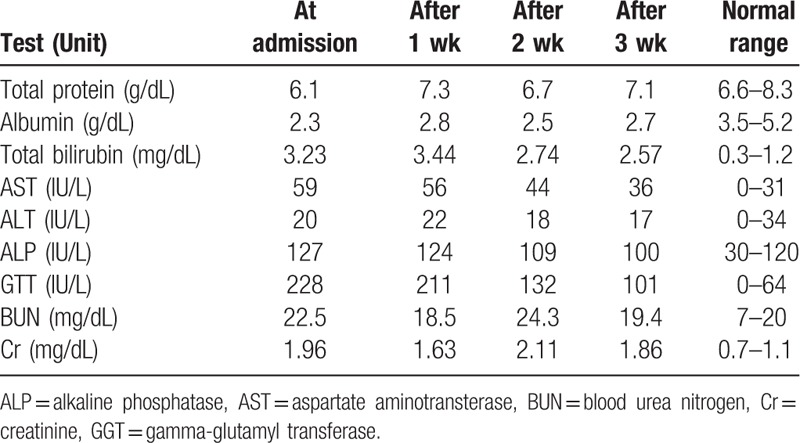
Changes of biochemical analysis.

## 4 Therapeutic intervention

The treatment goal was to alleviate the lower limb edema and improve liver function. We decided to use two THMs: Gwack Rhyung Tang (GRT) twice a day and Chunggan extract (CGX) three times a day. GRT is a THM for edema,^[10]^ and is composed of 148 g of 16 herbs (Table 2). It was prepared by boiling the herbs in 1000 mL distilled water for approximately 3 hours until the volume was reduced to 140 mL. CGX (Samik Pharmacy, Seoul, Korea) is a THM extract comprising 13 herbs and its name means “cleaning liver.” It has been in South Korea since 2001 for patients with liver diseases, such as alcohol-induced and toxic liver injuries and liver fibrosis.^[11–13]^ Our patient continued to receive his existing medications: amlodipine 5 mg, furosemide 40 mg, spironolactone 75 mg, pioglitazone 16.53 mg, teneligliptin 31 mg, glimepiride 8 mg, rifaximin 600 mg, ursodeoxycholic acid 400 mg, lactitol 40 g, ferrous sulfate 256 mg, and esomeprazole magnesium 20 mg daily.

**Table 2:**
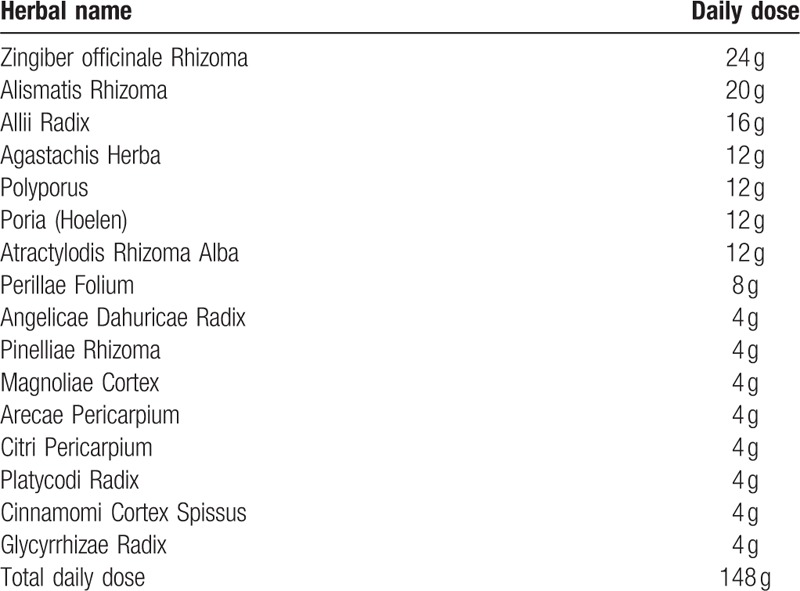
Components of Gwack Ryung Tang (GRT).

**Figure 3:**
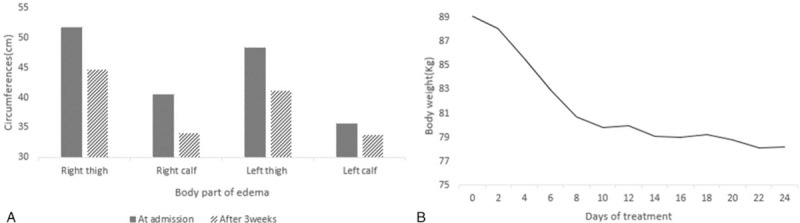
(A) Changes in thigh/calf circumferences (B) Changes in body weight. After 25 days of treatment, right thigh and calf remarkably slimmed down to 86% and 83%, respectively with obvious body weight loss.

A rapid response to THM treatment was confirmed through the loss of body weight and discernible changes of lower limb edema after 3 weeks (Fig. 1B). Over 25 days, he lost 11 kg and the circumferences of the right thigh and calf decreased by 14% and 17%, respectively (Fig. 3). On December 15, we reduced the dose of the diuretic furosemide from 40 mg to 20 mg per day and observed that the edema did not worsen. Biochemical analyses revealed improvements in liver and kidney function. Bilirubin levels decreased from 3.23 to 2.57 mg/dL (Table 1). The patient was discharged after 25 days of THM treatment and remained in good condition at the 3-week follow-up. During the treatment, no specific adverse effects were observed.

## 5 Discussion

We reported a patient with refractory lower limb edema that did not respond to diuretics for more than 7 months. The patient was treated with GRT and CGX for 25 days. The circumferences of both thighs and calves decreased remarkably and the biochemistry parameters improved. There was no aggravation at the 3-week follow-up.

The recommended treatment of edema due to cirrhosis is to start diuretic therapy immediately with a sodium-restricted diet and bed rest.^[7,14]^ While loop diuretics are usually the drug of choice for edema, the first-line therapy for cirrhosis is spironolactone, an aldosterone antagonist in renal collecting tubules, alone or along with furosemide (a loop diuretic). The recommended dose is to start spironolactone at 100 mg/day and furosemide at 40 mg/day.^[15]^ The spironolactone can be increased to 400 mg/day,^[16]^ carefully based on the patient's daily weight loss and serum levels of potassium, sodium, and creatinine. Despite such therapy, refractory hepatic edema can persist and the high doses of medication can cause hepatotoxicity or nephrotoxicity.

In this case, our treatment targeted reducing the lower limb edema and treating the underlying liver cirrhosis. GRT is a THM formula which was first described in traditional medical text, Uihakipmun, published by Lee Chun in 1575. Some of its components are also used to make Oryeongsan, a well-known THM formula for edema.^[10,17,18]^ Each of the herbs used in GRT had diuretic effects. Representative herbs with diuretic effects in GRT include *Polyporus* sp.,^[19–21]^*Poria cocos*^[22]^ and *Alismatis orientale* rhizome.^[23]^ The diuretic mechanism of *Polyporus* involves the downregulation of aquaporin-2 and vasopressin type 2 receptor.^[19]^ Ergone, a compound found in *Polyporus*, is an aldosterone antagonist that does not act directly on Na^+/^K^+^ ion channels.^[21]^*Poria cocos* suppresses arginine vasopressin levels and downregulates mRNA expression of vasopressin type 2,^[22]^ similar to the diuretic mechanism of *Polyporus*. In rats, *Alisma* rhizome extract increases urine output with obvious increases in Na^+^, Cl^–^, and K^+^ excretion, which implies that it interferes with the ion transport carrier in the thick ascending limb of the loop, similar to furosemide.^[23]^ Diuretic resistance has not been studied in any herbal medicines. Because the basic treatment strategy for refractory edema combines different types of diuretic, we speculate that the success of GRT resulted from the synergetic effects of the diuretic herbal medicines. Considering that diuretics can be used for edema caused by several underlying diseases, such as heart failure, renal nephrotic syndromes, and liver cirrhosis, GRT can also be prescribed for edema caused by other diseases. THM treatment strategy is decided based on the patient's symptoms and pathological patterns rather than on the basis of the underlying diseases. Therefore, GRT can be an alternative for edema which is characterized as ’Damp’ pathological pattern.

CGX has received attention as a complementary remedy for patients with chronic liver disorders based on the results of experimental studies.^[11–13]^ One of the constituents of CGX is *Artemisia capillaris*, a well-known essential herb in liver diseases with antioxidant, anti-steatotic, anti-inflammatory, antiviral, choleretic, and anti-fibrotic effects.^[24]^

The short follow-up period and lack of a control group are study limitations. However, the success of the THMs at reducing the edema and improving liver function is truly inspiring. Further studies of the therapeutic effects of THMs and clinical trials are warranted.

## Author contributions

**Conceptualization:** Gayoung Lee, Jung-Hyo Cho, Namhun Lee.

**Supervision:** Namhun Lee.

**Writing – original draft:** Gayoung Lee.

**Writing – review & editing:** Jung-Hyo Cho, Chang-Gue Son, Namhun Lee.

Gayoung Lee orcid: 0000-0002-6242-4170.
